# Changes in Bacterial and Fungal Communities across Compost Recipes, Preparation Methods, and Composting Times

**DOI:** 10.1371/journal.pone.0079512

**Published:** 2013-11-21

**Authors:** Deborah A. Neher, Thomas R. Weicht, Scott T. Bates, Jonathan W. Leff, Noah Fierer

**Affiliations:** 1 Department of Plant and Soil Science, University of Vermont, Burlington, Vermont, United States of America; 2 Department of Plant Pathology, University of Minnesota, Minneapolis, Minnesota, United States of America; 3 Cooperative Institute for Research in Environmental Sciences, University of Colorado, Boulder, Colorado, United States of America; 4 Department of Ecology and Evolutionary Biology, University of Colorado, Boulder, Colorado, United States of America; Washington State University, United States of America

## Abstract

Compost production is a critical component of organic waste handling, and compost applications to soil are increasingly important to crop production. However, we know surprisingly little about the microbial communities involved in the composting process and the factors shaping compost microbial dynamics. Here, we used high-throughput sequencing approaches to assess the diversity and composition of both bacterial and fungal communities in compost produced at a commercial-scale. Bacterial and fungal communities responded to both compost recipe and composting method. Specifically, bacterial communities in manure and hay recipes contained greater relative abundances of Firmicutes than hardwood recipes with hay recipes containing relatively more Actinobacteria and Gemmatimonadetes. In contrast, hardwood recipes contained a large relative abundance of Acidobacteria and Chloroflexi. Fungal communities of compost from a mixture of dairy manure and silage-based bedding were distinguished by a greater relative abundance of Pezizomycetes and Microascales. Hay recipes uniquely contained abundant *Epicoccum*, *Thermomyces*, *Eurotium*, *Arthrobotrys*, and *Myriococcum*. Hardwood recipes contained relatively abundant Sordariomycetes. Holding recipe constant, there were significantly different bacterial and fungal communities when the composting process was managed by windrow, aerated static pile, or vermicompost. Temporal dynamics of the composting process followed known patterns of degradative succession in herbivore manure. The initial community was dominated by Phycomycetes, followed by Ascomycota and finally Basidiomycota. Zygomycota were associated more with manure-silage and hay than hardwood composts. Most commercial composters focus on the thermophilic phase as an economic means to insure sanitation of compost from pathogens. However, the community succeeding the thermophilic phase begs further investigation to determine how the microbial dynamics observed here can be best managed to generate compost with the desired properties.

## Introduction

Municipalities, industry and agricultural farms are generating substantial amounts of organic wastes. These wastes not only strain landfill space, but also pose serious threats to the environment. Compostable materials (including paper, food wastes, and grass clippings) comprised 62% (155 million tons) of this waste stream in the US [1]. Composting represents an important solution for a more sustainable management of organic waste. Not only does composting remove waste, it can effectively convert the waste into a nutrient-rich organic amendment for a variety of agricultural, horticultural or landscaping applications. Countries, states, and municipalities are increasingly enacting legislation and regulation to promote the diversion of organics from solid waste disposal facilities to recycling and composting.

Composting is a controlled aerobic process that degrades organic waste to stable material, with the resident microbial community mediating the biodegradation and conversion processes. There are three distinct successional phases driving chemical and microbial changes through time, phases that are determined primarily by changes in temperature [2]: mesophilic phase (moderate temperatures rising to ∼45°C), thermophilic phase (high temperatures peaking at ∼70°C), curing phase (cooling to ambient temperature). Compost recipes can vary widely. For example, carbon sources can include straw, paper, woodchips, sawdust, or bark; whereas nitrogen sources can include animal manures, sewage sludge, and/or municipal solid waste. Large-scale commercial composting requires a high-temperature phase designed to facilitate the removal of human and plant pathogens. The primary types of commercial composting methods are windrow, aerated static pile, and vermicomposting. Although vermiculture does not inherently include a thermophilic phase [3], [4], material can be pre-composted through aerated static piles and windrow to remove substances toxic to earthworms, inactivate plant seeds and remove human and plant pathogens. Generally, we have a poor understanding of the biological dynamics that occur during the composting process and there are no current regulations or guidelines that define desirable microbiological properties of compost.

Microbial communities found in compost have not been well characterized and relatively few studies describe both their bacterial and fungal diversity even though both groups are likely important mediators of the composting process. Most studies have utilized culture-based methods [2], [4], [5], [6], [7] that are known to only capture a small portion of the microbial diversity found in environmental samples [8]. While a few studies have employed cultivation-independent approaches, a comprehensive perspective on compost microbial dynamics is still lacking because these studies have focused on only one of the three compost phases [9], [10], [11], [12], [13], [14] or have used fingerprinting techniques that offer limited taxonomic resolution [15], [16], [17], [18], [19], [20], [21]. The few available high-throughput sequencing studies have focused primarily on bacteria [22], [23], [24], [25] even though fungi likely play an important role in the compost process and may enhance the quality of compost. Overall, we still have a limited understanding of how microbial communities are influenced by different composting recipes or methods, or the phases of heating and cooling that occur during the compost process.

This study represents a comprehensive assessment of both bacteria and fungi associated with compost using high-throughput sequencing on the Illumina MiSeq platform. We investigated the influence of composting recipe and process on the structure of microbial communities, and how communities change through time when compost is produced on a commercial-scale.

## Methods

### Field site and sampling

To insure validity and applicability of results, all compost recipes were produced at a commercial compost production facility at Highfields Center for Composting (HCC; Hardwick, Vermont). All recipes contained 25% manure-silage from the same source, and all composting processes included a thermophilic phase. For all experiments, a sample is defined as a composite of 10 subsamples collected at random depths from a given pile that are mixed to be representative of a pile. Samples were frozen at −20°C until processed.

### Experiment 1. Compost recipes

Three recipes contained manure from the same source with varying carbon sources were prepared and composted using the aerated static pile process. Recipe one was 100% manure-silage from a typical Vermont dairy barn, with a C∶N ratio of 17∶1, as a control. Recipe two used hay as a carbon source, and was mixed in a 3∶1 ratio (volume basis) with manure/silage resulting in a C∶N ratio of 23∶1. Recipe three used hardwood as a carbon source mixed in a 5∶5∶3 ratio of manure/silage∶hardwood bark: softwood shavings resulting in a C∶N ratio of 34∶1. During the early curing phase, compost was delivered to two farm locations where it continued to cure for three months prior to sampling. In total, eight samples were taken from each of the three recipes for a total of 24 samples. Hereafter, the recipes will be referred to as ‘manure-silage’, ‘hay’, and ‘hardwood’, respectively.

### Experiment 2. Composting process

A standard commercial recipe was used in each of the three composting processes: windrow, aerated static pile, vermicompost. This recipe was comprised of 20% food residuals, 10–15% 2.5 cm woody material (e.g., hardwood bark and mixed wood chips), 10% hay, up to 5% shredded paper, up to 2% dry sawdust or shavings, and 50–60% mixed livestock manures (e.g., horse, cow, heifer, calf) mixed with various bedding materials (e.g., straw and hay). Four replicate samples were taken per treatment, for a total of 12 samples. Total genomic DNA was extracted from two subsamples for each of two piles per composting process. Windrow, aerated static pile, and vermicompost samples were collected after curing, i.e., 9, 6, and 7 months of composting, respectively.

#### Windrow

Windrow involves placing a mixture of organic waste materials into long, narrow piles on a composting pad which are turned frequently [3]. Piles were mixed with a bucket loader and were capped with manure/bedding to meet Vermont and National Organic Standards (NOS) Board regulations (www.ams.usda.gov/nop) and the piles were managed to maintain a temperature between 55–77°C for a minimum of 15 days and turned with a bucket loader a minimum of five times to ensure all materials have been subjected to the minimum temperature requirements.

#### Aerated Static Pile (ASP)

ASP systems force air throughout the pile and does not require turning once the pile has been formed, thus allowing for larger piles to be produced [3]. Piles were mixed with a bucket loader, placed in a three-sided ASP bay, and capped with manure/bedding. Each ASP pile was built to a height of 2 to 2.5 m after settling. Initial piles were 57–76 m^3^ when placed in ASP bays. Piles were aerated in place with an in-floor air delivery system that uses a 20 cm layer of wood chips between the duct-work and pile to evenly distribute air. Blower fans were managed with speed control and timers, to meet ‘Process to Further Reduce Pathogens’ (PFRP) requirements as dictated by Vermont and NOS regulations. Briefly, piles were aerated in the ASP system for 3–6 weeks so the pile attained temperatures of 55°C for a minimum of three days. Piles were then re-stacked on composting pads, and turned regularly to continue composting.

#### Vermicompost

Vermicompost is a mesophilic process that employs earthworms to stabilize organic residues [4]. Material entering the Continuous-Flow Worm Reactor was taken from piles on the composting pad, after they have been through the ASP procedure (outlined above), and re-stacked on the pads. Material had already met PFRP, and was generally four to six weeks old. Fresh material (0.76 m^3^) was spread out weekly in a 3.8 cm layer on top of the bed, where it was allowed to continue to decompose, and be consumed by earthworms (*Eisenia fetida*). The bed was 1.52 m wide, 12.19 m long, and 0.6 m deep. The worm bed was housed in an indoor, heated room, and the compost temperature was 21–27°C. The compost remained in the worm bin for 60 to 90 days, the time it took for the fresh compost to be decomposed and move downward and out of the bottom of the bed.

### Experiment 3. Changes in microbial communities during the composting process

A standard commercial recipe of HCC was composted through windrow piles, aerated static pile, and vermicompost. Samples were collected on 20 September 2012 and represent various ages throughout the thermophilic and curing phases of the compost process for windrow, aerated static pile, and vermicomposting. Duplicate samples were analyzed at each time point for each composting method yielding a total of 24 samples.

### DNA extraction, PCR amplification, sequencing

Genomic DNA was extracted using the MoBio PowerSoil™ kit (MoBio, Carlsbad, CA, USA) according to the manufacturer's instructions following the method described in Lauber et al. [26]. PCR amplification of the 16S rRNA gene (for bacteria and archaea) or the internal transcribed spacer region (ITS1) of the nuclear ribosomal RNA gene (for fungi) followed the approach described in Fierer et al. [27]. Briefly, each sample was amplified in triplicate, and amplicons were composited together in equimolar concentrations prior to sequencing. PCR reactions contained 13 µL PCR-grade water, 10 µL 5 Prime Hot Master Mix, 0.5 µL each of the forward and reverse primers (10 µM final concentration), and 1.0 µL genomic DNA (diluted 1∶10 with PCR-grade water). Reactions were held at 94°C for 3 min to denature the DNA, with amplification proceeding for 35 cycles at 94°C for 45 s, 50°C for 60 s, and 72°C for 90 s; a final extension of 10 min at 72°C was added to ensure complete amplification. For the bacterial and archaeal analyses, the PCR primers (515f/806r) targeted the V4 region of the 16S rRNA gene [28]. For the fungal analyses, we used PCR primers (ITS1-F/ITS2) to amplify the ITS1 spacer [29]. Both primer pairs contained 12-bp barcodes unique to each sample and the appropriate adapters to permit sequencing on the Illumina MiSeq platform [28], [30].

### Data analysis

Quality filtering, assignment of sequences to samples based on their barcodes, and clustering of sequences into operational taxonomic units (OTUs) was done following the standard QIIME pipeline [31] with sequence data quality-filtered as described previously. OTUs were determined using an open reference-based approach that implements reference-based clustering followed by *de novo* clustering using the UCLUST algorithm [32]. Clustering was conducted at the 97% similarity level using pre-clustered versions of the October 2012 Greengenes database (for 16S rRNA) [33] and November 2012 UNITE database (fungal ITS gene) [34] for the sequence reference set. Fungal ITS sequence processing followed the procedure outlined in McGuire et al. [30]. Sequences were assigned to taxonomic groups using the RDP classifier [35]. To keep sequencing depth consistent across all samples, the sequence data were rarified by randomly subsampling 2,000 and 100 reads per sample before downstream analyses of the 16S and fungal ITS rRNA datasets, respectively. Amplicon sequences were deposited in the public EMBL-EBI database (http://www.ebi.ac.uk/) and may be accessed using the accession numbers, ‘ERP003625’ and ‘ERP003626’ for the 16S and ITS sequences, respectively.

### Statistical analysis

We used the PRIMER v.6 software package (PRIMER-E, Plymouth, WA, USA) [36] for the calculation of pair-wise differences in community composition (Bray-Curtis distances) and the subsequent analyses of the pair-wise dissimilarity matrices via principal coordinate analysis and permutational multivariate analysis of variance (PERMANOVA). We used PERMANOVA to assess the effects of compost type and process type on the composition of the bacterial and fungal community compositions. For compost type, we included the farm identity as a random factor in our model to account for variation between farms. Differences in the relative abundance of specific taxa across recipe and methods were determined using multiple Kruskal-Wallis tests in *R* [37] and applying false discovery rate corrections to *p*-values to account for the multiple comparisons. Tests were only performed for the more abundant taxa (those with median relative abundances greater than 1.0% in any of the recipes or processes).

## Results

We obtained a total of 799,030 and 35,280 150-bp quality-filtered 16S and fungal ITS rRNA gene sequences across all samples, respectively. The number of 16S rRNA sequences obtained per sample varied from 770 to 13,390 (median = 5,398), and the number of fungal ITS sequences per sample varied from 51 to 663 (median = 215).

### Compost recipes

**Figure 1:**
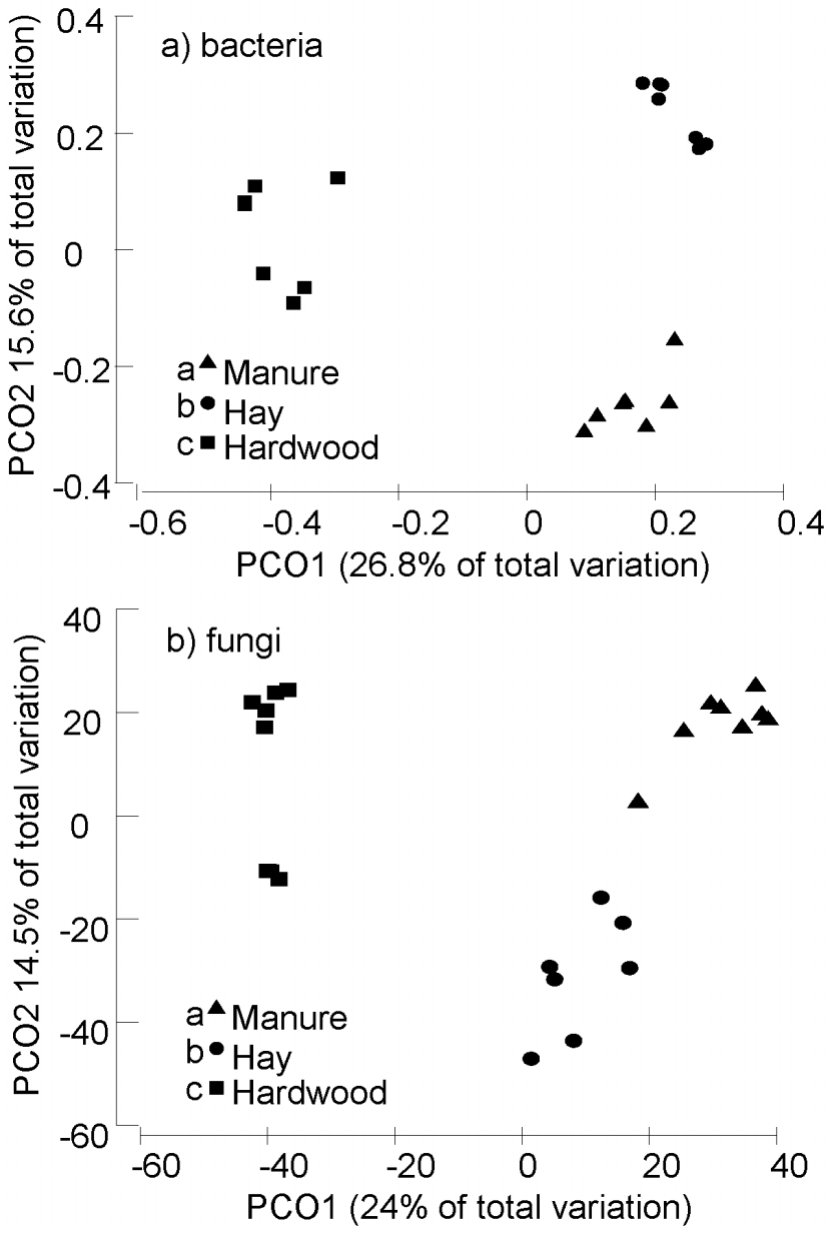
Principal coordinates analysis biplot for a) bacterial and b) fungal communities in three compost recipes (triangle: manure-silage, circle: hay, square: hardwood), *n* = 7 per treatment. Permutational multivariate analysis of variance indicated that differences between communities were highly significant (*p*≤0.001). Contrasting superscripts indicate that treatments are significantly different (*p*≤0.05).

All pair-wise comparisons of recipes contained unique communities of bacteria (p_(perm)_ = 0.001)and fungi (p_(perm)_ = 0.001). There was far more variability in bacterial (Table S1) and fungal community composition between compost recipes than across replicate samples collected from the same recipe (Figure 1).

Proteobacteria and Bacteroidetes were the most abundant bacterial phyla among compost recipes (Table 1). Within Proteobacteria, the γ-proteobacteria was more abundant than α-proteobacteria, β-proteobacteria, and δ-proteobacteria. Archaea were rare (median 1.2% of reads for all samples), comprised mostly of Crenarchaeota and Euryarchaeota. Similarly, manure and hay contained greater relative abundances of Firmicutes than hardwood. Uniquely, hay contained more Actinobacteria and Gemmatimonadetes. In contrast, hardwood contained a greater relative abundance of Acidobacteria and Chloroflexi.

**Table 1:** Mean ± 1 SD (*n* = 8) of dominant bacterial phyla and sub-phyla, expressed as percentage of sequences in cured manure, hay and hardwood compost recipes.

Taxon	Manure	Hay	Hardwood
Acidobacteria^***^	1.2±0.7	0.7±0.1	7.4±2.7
Actinobacteria*	4.9±2.0	9.9±3.8	6.8±2.0
Bacteroidetes*	27.0±4.8	27.7±2.7	21.0±3.6
Chloroflexi^***^	5.5±3.9	2.3±0.6	11.8±8.5
Firmicutes**	5.2±2.7	6.8±1.6	1.5±0.5
Gemmatimonadetes**	1.9±0.8	4.4±0.6	2.9±0.8
Planctomycetesn.s.	2.6±1.4	1.8±0.3	3.0±0.7
α-Proteobacterian.s.	7.8±1.6	6.2±2.4	6.3±1.7
β-Proteobacterian.s.	7.0±3.7	4.1±0.7	4.0±1.9
δ-Proteobacterian.s.	7.0±1.7	7.0±2.3	7.2±1.5
γ-Proteobacteria*	12.5±5.9	13.5±1.5	9.2±2.8
Verrucomicrobia^***^	1.7±1.0	1.5±0.3	4.6±1.1

False Discovery Rate (FDR) *p*-values from Kruskal-Wallis test,

n.s. : *p*_FDR_>0.05,

* :0.01<*p*_FDR_<0.05,

** : 0.001<*p*_FDR_<0.01.

Ascomycota was the most abundant fungal phylum among recipes, about nearly two-fold more abundant than the Basidiomycota (Table 2). Basidiomycota were represented by Agaricomycetes and two undefined taxonomic classes. Manure-silage was distinguished by containing the greatest abundance of Pezizomycetes (including *Ascobolus*) and Microascales. Uniquely, hay contained greater abundances of *Epicoccum*, *Thermomyces*, *Eurotium*, *Arthrobotrys*, and *Myriococcum* (Table 2). Manure-silage and hay, but not hardwood, contained Chytridomycota and Zygomycota with hardwood containing greater abundances of Sordariomycetes.

**Table 2:** Mean ± 1 SD of fungal genera, expressed as percentage of sequences classified to phylum level in cured manure, hay, and hardwood compost recipes.

					*n* = 8	*n* = 7	*n* = 8
Phylum	Class	Order	Family	Genus	manure	hay	hardwood
**Ascomycota** ns					**61.5±16.4**	**55.9±14.7**	**69.4±22.6**
	Dothideomycetes*	Pleosporales*	Pleosporaceae**	*Epicoccum* a **	0±0	2.1±1.5	0.3±0.8
	Eurotiomycetes**	Eurotiales**	Unknown^b^^, ^ ns	*Thermomyces* ns	0±0	2.7±3.7	0±0
		Eurotiales**	Trichocomaceae**	*Eurotium* **	0±0	2.0±2.0	0±0
	Orbiliomycetes*	Orbiliales*	Orbiliaceae*	*Arthrobotrys* c *	0.4±0.9	6.1±7.0	0±0
	Pezizomycetes*	Pezizales*	Ascobolaceae*	*Ascobolus* *	9.3±12.3	2.4±3.7	0±0
		Unknown*	Unknown*	Unknown*	3.2±3.1	1.1±1.3	0±0
	Sordariomycetes*	Hypocreales*	Unknown**	*Acremonium* *	0±0	1.4±1.1	0±0
		Microascales**	Microascaceae**	*Pseudallescheria* ns	2.7±3.7	0.2±0.4	0±0
		Microascales**	Microascaceae**	*Scedosporium* d *	0.2±0.5	7.7±16.1	0±0
		Microascales**	Microascaceae**	Unknown**	14.1±12.9	1.8±2.5	0±0
		Microascales**	Unknown**	Unknown**	7.7±7.4	0.5±1.2	0±0
		Unknown**	Unknown**	Unknown**	14.9±10.6	7.0±4.5	54.2±16.7
		Sordariales*	Lasiosphaeriaceae^ns^	*Zopfiella* ns	0.3±0.9	1.5±2.8	3.4±5.4
		Sordariales*	Lasiosphaeriaceae^ns^	Unknown**	0±0	0±0	2.3±1.8
		Sordariales*	Unknown**	Unknown**	0±0	0.2±0.4	2.8±2.6
	Unknown^ns^	Unknown^ns^	Unknown^ns^	Unknown^ns^	4.9±3.4	5.3±2.6	5.4±4.4
**Basidiomycota** ns					**29.0±15.8**	**35.3±16.7**	**30.3±22.9**
	Agaricomycetes*	Agaricales^ns^	Psathyrellaceae^ns^	*Coprinellus* ns	0.3±0.9	0±0	6.6±9.6
		Agaricales^ns^	Psathyrellaceae^ns^	*Coprinus* ns	3.2±6.9	0±0	1.0±2.1
		Agaricales^ns^	Psathyrellaceae^ns^	Unknown*	3.4±3.9	1.5±2.1	0±0
		Corticiales^ns^	Corticiaceae^ns^	Unknown^ns^	0±0	1.3±1.4	2.5±3.7
		Unknown**	Unknown**	*Myriococcum* e **	0±0	9.8±9.7	0±0
		Unknown^ns^	Unknown^ns^	Unknown^ns^	0.7±1.0	18.0±15.5	18.4±24.6
	Unknown**	Unknown**	Unknown**	Unknown**	16.8±11.0	0±0	0±0
	Unknown*	Unknown*	Unknown*	Unknown*	3.2±1.6	2.8±2.8	0.8±1.3
**Chytridiomycota** *					**6.7±6.7**	**5.0±5.6**	**0±0**
	Chytridiomycetes*	Spizellomycetales^ns^	Spizellomycetaceae^ns^	*Gaertneriomyces* ns	0.3±0.8	1.6±4.2	0±0
		Unknown*	Unknown*	Unknown*	4.8±6.6	3.4±3.3	0±0
**Zygomycota** *					**2.9±2.2**	**3.8±3.2**	**0.3±0.8**
	Incertae_sedis*	Harpellales*	Legeriomycetaceae*	*Smittium* f *	1.3±1.5	0.3±0.6	0±0
		Mortierellales^ns^	Mortierellaceae^ns^	*Mortierella* ns	1.1±1.6	1.1±2.9	0±0
		Mucorales^ns^	Mucoraceae^ns^	*Mucor* ns	0±0	2.1±2.9	0±0

False Discovery Rate (FDR) *p*-values from Kruskal-Wallis test,

n.s. : *p*_FDR_>0.05,

* :0.01<*p*_FDR_<0.05,

** : 0.001<*p*_FDR_<0.01.

a : rank order of *Epicoccum* species abundance: *E.* sp_CHTAM7, *E.* sp_TMS_2011.

b represents a) a sequence from an undescribed taxon, b) from an environmental sequence were the organism was not identified, or c) a sequence matches a described species that is not represented in the reference database.

c : rank order of *Arthrobotrys* species abundance: *A. amerospora*>*A. flagrans*>*A. oligospora*.

d : rank order of *Scedosporium* species abundance: *S. prolificans*>*S. aurantiacum*>*S. apiospermum*.

e : dominant species: *Myriococcum thermophilum*.

f : rank order of *Smittium* species abundance: *Smittium* sp.>*S. orthocladii*.

### Compost process

**Figure 2:**
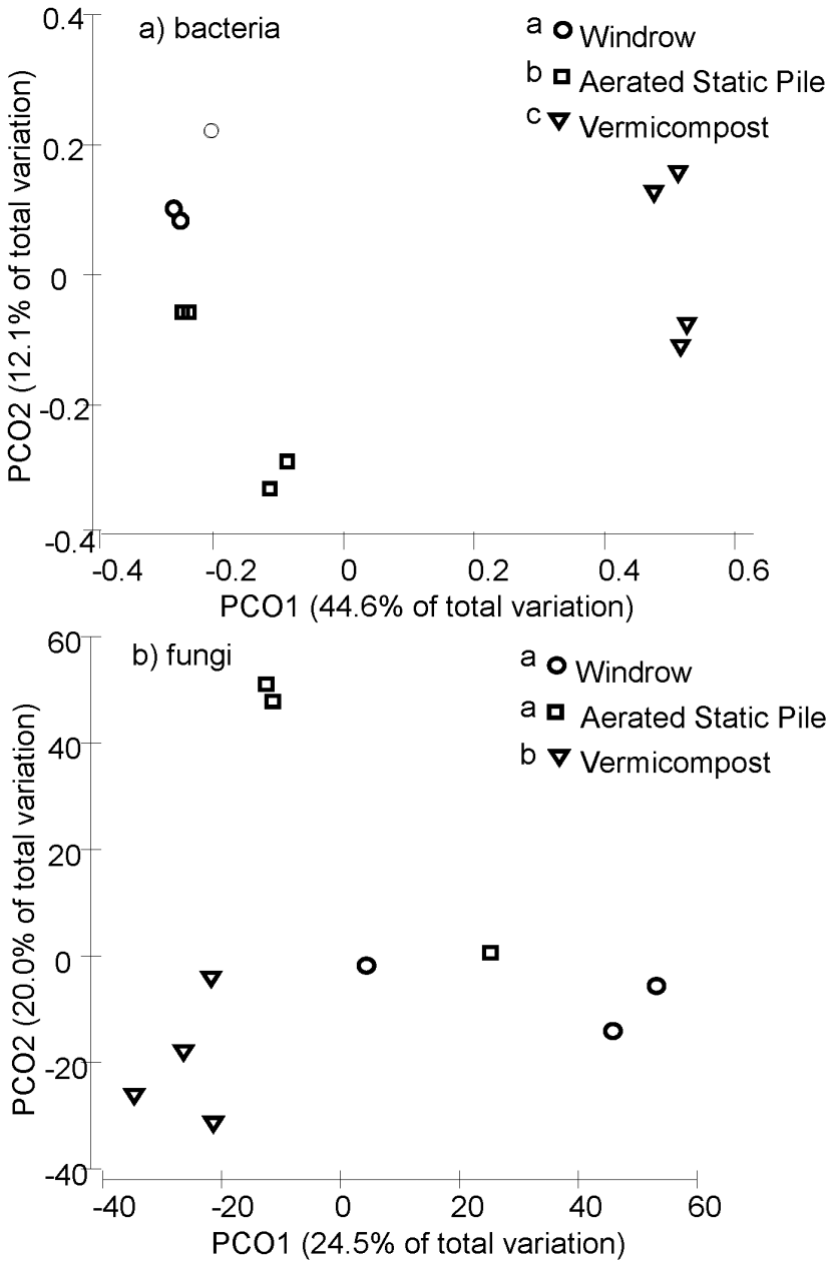
Principal coordinates analysis biplot for a) bacterial and b) fungal communities in the end product of three compost processes (circle: windrow, square: aerated static pile, inverted triangle: vermicompost), *n* = 4 per process. Permutational multivariate analysis of variance indicated that differences between communities were highly significant (*p*≤0.001). Contrasting superscripts indicate that treatments are significantly different (*p*≤0.05).

Holding recipe constant, bacterial (p_(perm)_ = 0.002, Fig. 2A) and fungal (p_(perm)_ = 0.003, Figure 2B) communities varied according to the type of managed compost process (Table S2). The compost prepared by ASP and windrow harbored bacterial and fungal communities that were more similar to one another than to the vermicompost-treated compost (Figure 2).

Bacterial communities in the windrow method were characterized by having a greater relative abundance of Chloroflexi and Chlorobi (Table 3). In contrast, the product of the vermicompost process contained relatively abundant Bacteroidetes, γ-Proteobacteria, and Verrucomicrobia. Fungal communities of windrow were dominated by Sordariomycetes, *Acremonium*, and an unclassified group in the Basidiomycota (Table 4). ASP was distinguished by a greater relative abundance of an unclassified family of Pezizales. Vermicompost contained the greatest relative abundance of *Arthrobotrys*, Microascaceae, *Zopfiella*, Agaricomycetes, and *Mortierella*.

**Table 3:** Mean ± 1 SD (*n* = 4) of total sequences classified as bacteria in a common recipe processed by windrow, aerated static pile or vermicompost.

	Windrow	Aerated Static Pile	Vermicompost
Bacteroidetes*	21.5±4.6	16.3±0.7	29.4±10.5
Chlorobi*	2.9±1.1	1.0±0.2	0.2±1.1
Chloroflexi*	19.8±3.8	8.0±1.2	2.4±7.3
γ-Proteobacteria∧	10.3±1.3	14.6±1.2	16.5±9.7
Verrucomicrobia∧	2.0±0.8	1.5±1.3	4.2±1.2

Values are expressed as percentages.

* : *p*≤0.05 false discovery rate (adjusted) from KW and unadjusted P-values.

∧ : *p*≤0.05 for unadjusted *P*-value, but ≤0.1 for false discovery rate (adjusted).

**Table 4:** Mean ± 1 SD of fungal ITS sequences classified to phylum level in a common recipe processed by windrow, aerated static pile or vermicompost.

					*n* = 3	*n* = 3	*n* = 4
Phylum	Class	Order	Family	Genus	Windrow	Aerated Static Pile	Vermicompost
**Ascomycota**					**81.6±9.9**	**87.5±7.1**	**75.0±15.3**
	Orbiliomycetes^ns^	Orbiliales^ns^	Orbiliaceae^ns^	*Arthrobotrys* ns	6.4±10.1	9.7±9.4	15.4±12.5
	Pezizomycetes^ns^	Pezizales^ns^	Pezizaceae^ns^	Unknown^ns^	0±0	2.6±4.5	8.2±14.1
		Pezizales^ns^	unidentified^ns^	Unknown^ns^	0±0	36.2±31.9	0.9±1.7
	Sordariomycetes^ns^	Hypocreales^ns^	Unknown^ns^	*Acremonium* ns	10.4±18.0	0±0	0±0
		Microascales^ns^	Microascaceae^ns^	Unknown^ns^	0±0	1.3±1.3	15.9±6.1
		Sordariales^ns^	Lasiosphaeriaceae	*Zopfiella* ns	0±0	0±0	4.1±5.1
		Sordariales^ns^	Unknown^ns^	Unknown^ns^	0.8±1.5	2.1±3.6	1.2±1.4
		Unknown^ns^	Unknown^ns^	Unknown^ns^	44.9±27.7	18.9±27.0	5.1±4.4
	Unknown^ns^	Unknown^ns^	Unknown^ns^	Unknown^ns^	11.7±10.1	10.8±5.7	5.3±2.5
**Basidiomycota**					**13.3±9.3**	**7.6±3.0**	**14.9±16.0**
	Agaricomycetes^ns^	Agaricales^ns^	Unknown^ns^	Unknown^ns^	0.4±0.7	0±0	9.8±16.4
	Unknown^ns^	Unknown^ns^	Unknown^ns^	Unknown^ns^	4.4±6.1	1.7±2.0	1.2±1.5
**Zygomycota**					**4.4±4.1**	**4.5±4.3**	**9.0±3.7**
	Insertae_sedis^ns^	Mortierellales^ns^	Mortierellaceae^ns^	*Mortierella* ns	3.9±3.4	3.0±3.1	6.1±6.6

Values are expressed as percentages.

False Discovery Rate (FDR) *p*-values from Kruskal-Wallis test,

n.s. : *p*_FDR_>0.05.

Unknown can represent other or unidentified.

### Dynamics of composting process

**Figure 3:**
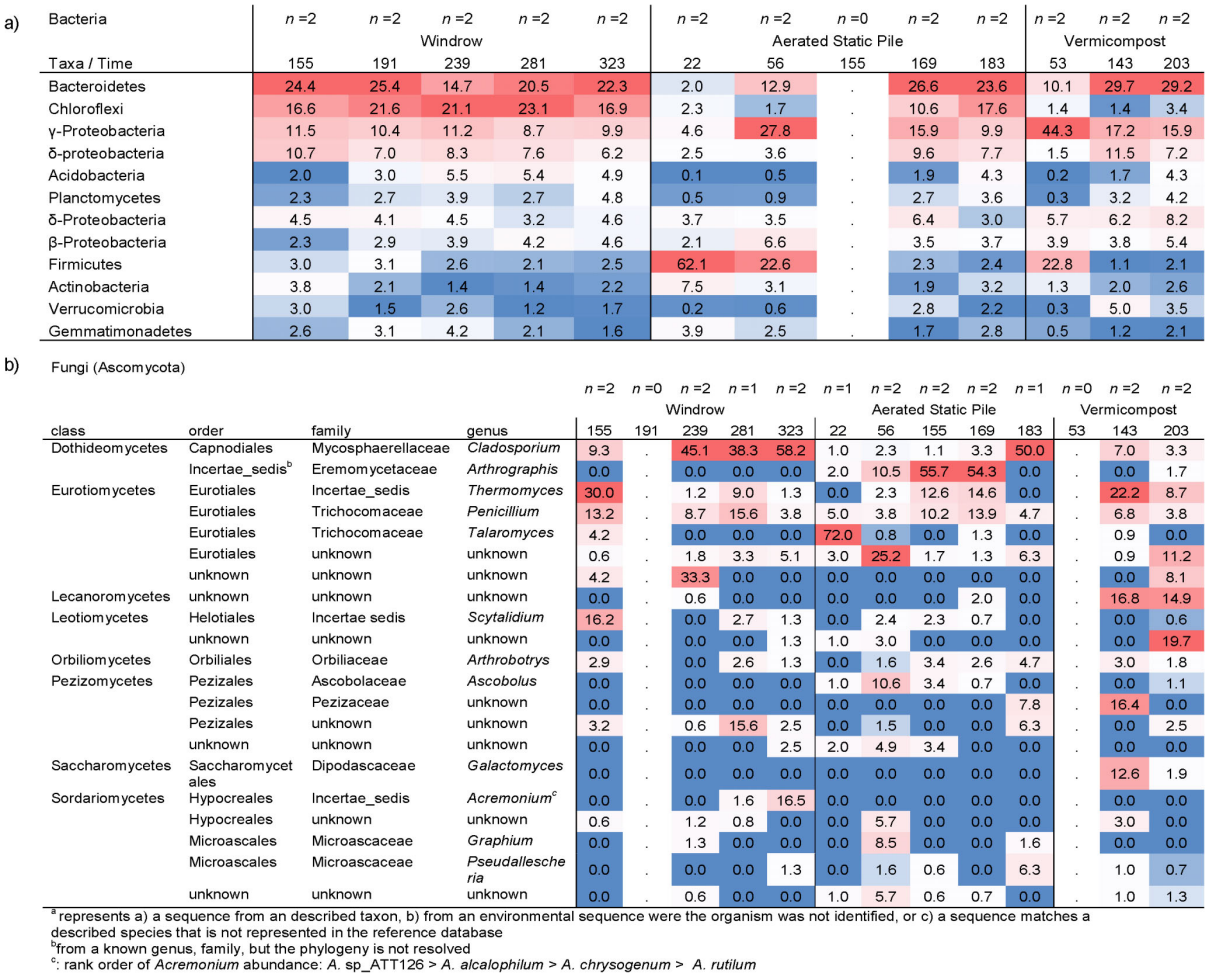
Heat map illustrating changes in A) bacterial and B) fungal composition through time for the same recipe composted by three processes: windrow, aerated static pile or vermicompost. All fungi illustrated are ascomycota. Time is expressed as days of decomposition. The thermophillic phase occurred prior to sampling in windrow, days 22–56 for aerated static pile, and day 53 for vermicompost. Units illustrated as mean percentages of total sequences (bacteria) and percentage of taxa classified to phylum (fungi). Dots represent missing samples. Each column is colored so that taxa with high relative abundance are red, intermediate abundances are white and low abundances are blue.

The temporal shifts in bacterial and fungal communities during the composting process were influenced primarily by whether the curing phase was managed as windrow, ASP or vermicompost (Figure 3). For the bacteria, Bacteroidetes varied by compost process through time (Figure 3A). Bacteroidetes were abundant following the thermophilic phase, but declined and subsequently increased in relative abundance as composting progressed in the windrow process but steadily increased through time in the ASP and vermicompost processes. The thermophilic phase of ASP and vermicompost was dominated by Firmicutes. The relative abundance of γ-Proteobacteria increased soon after the thermophilic phase and declined through time for all composting processes but to different extents. Chloroflexi were relatively abundant at the end of the thermophilic phase of ASP, fluctuated in abundance through time in windrow, and were uncommon in vermicompost.

Fungal community composition varied through time and in different trajectories for the three composting processes (Figure 3B). In windrow, *Thermomyces* and *Scytalidium* were the dominant fungi after the thermophilic phase and declined through time whereas *Cladosporium* increased through time (Table 3b). *Acremonium* was second to *Cladosporium* in dominance within the finished compost. Talaromyces dominated the thermophilic phase of ASP while *Arthrographis* and *Cladosporium* predominate later in the ASP process. Vermicompost had approximately equal abundances of *Thermomyces*, Pezizaceae, *Galactomyces*, and Lecanoromycetes with similar abundances of Lecanoromycetes remaining in finished compost, accompanied by Eurotiales and Leotimycetes.

## Discussion

As the most comprehensive assessment of compost bacterial and fungal communities conducted to date, this work provides unique insight into microbial dynamics across different compost recipes, preparation techniques, and through time as compost cures. The types of bacterial and fungal taxa found in compost with the high-throughput sequencing methods employed here are similar to previous studies that have used other approaches to describe microbial communities in composts of similar feedstock and/or process as in this study [2], [7], [10], [12], [23], [25], [38]. For example, we found large numbers of sequences for Bacteroidetes, Proteobacteria, *Acremonium*, *Ascobolus*, and *Mortierella*, taxa that have been commonly associated with compost. Furthermore, *Aspergillus*, *Penicillium*, *Mucor*, and *Alternaria* were present as common saprophytic fungi on food wastes [2], [4], [5].

### Compost recipe

There are distinct types of microbial communities in finished compost products that originate from different source materials. We found taxa similar to those reported for compost and its starting ingredients of manure, hay, and hardwood. For example, temporal dynamics of the composting process followed known patterns of ecological succession in herbivore manure [39]. The initial community was dominated by Phycomycetes, mostly Mucorales, such as *Mucor* and *Mortierella*, followed by ascomycota such as *Ascobolus* and *Chaetomium* spp., and finally basidiomycota such as *Coprinus* and *Stropharia* spp. [39]. Although manure- silage contained the greatest volume of manure, the other recipes also contained manure which explains why taxa commonly associated with animal feces (e.g., Bacteroidetes, Firmicutes, γ-Proteobacteria, *Chaetomium*, *Coprinis*, and *Ascobolus*) were found in all recipes [2], [19], [40]. Zygomycota in the Harpelles, Mortierellales, and Mucorales were associated more with manure-silage and hay than hardwood composts.

Fungi associated with tree bark were more commonly associated with hardwood compost, e.g., Sordariomycetes and Agaricomycetes. All recipes contained *Zopfiella* which typically arrives later in succession [41]. *Trichoderma*, *Alternaria*, and *Aspergillus* were not dominant on finished hardwood compost. To our knowledge, this is the first report of hardwood compost containing relatively abundant bacterial taxa within the Acidobacteria and Verrucomicrobia phyla, or of hay being a favorable habitat for Actinobacteria and Gemmatimonadetes. Acidobacteria, Gemmatimonadetes, and Chloroflexi have all been reported in waste water and sludge [42], [43], [44], but this is the first study to note their importance in the compost process. These taxa are notoriously difficult to culture and the ecological attributes of many members of these groups are not well-known.

### Compost process

Different methods of composting after the thermophilic phase affect the dynamics and resulting composition of bacterial and fungal communities (Figure 2). We expected Actinobacteria and Firmicutes from windrow and ASP, and more Chloroflexi, Acidobacteria, Bacteroidetes, and Gemmatimonadetes in vermicompost [10]. The dominance of Ascomycota in both windrow and vermicompost processes has been documented in both culture-based [4] and 454-pyrosequencing [38] studies. However, in contrast to reports based on culturable fungi, we did not find *Fusarium* species to be a magnitude of order greater in windrow than vermicompost, or for *Trichoderma* species (anamorph: *Hypocrea*, Hypocreaceae) to be present exclusively in windrow [4].

**Figure 4:**
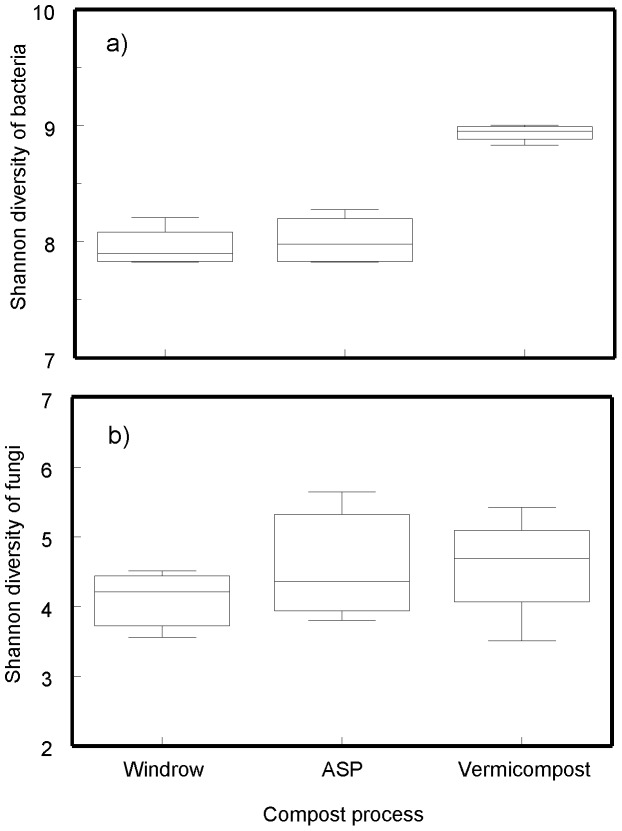
Shannon diversity of a) bacteria and b) fungal communities within a standardized recipe finished by windrow, aerated static pile (ASP) or vermicompost. Shannon diversity is computed as H′ = −Σ(p_i_ ln p_i_) where *p* represents the proportion of taxon *i* in the community. Box-whisker plots are illustrated.

Vermicompost had substantially different microbial communities when compared to those from ASP and windrow processes. These differences may be driven, in part, by differences in temperature regimes. Although vermicompost can be exclusively mesophilic, we inserted a thermophilic stage (ASP) prior to vermicomposting. We observed a greater diversity of bacteria in vermicompost than windrow and ASP (Figure 4). Our results support earlier reports that earthworms promote growth of bacteria [45] including Bacteroidetes, Verrucomicrobia, Firmicutes, and Proteobacteria [46]. There was also a trend for greater diversity of fungi in vermicompost and a relatively high abundance of fungi including *Mortierella* and *Arthrobotrys* supporting earlier reports [4], [47]. *Arthrobotrys* is a nematode-trapping fungus and been reported to be associated with earthworm inhabited soils [48]. In addition to the partially decomposed compost, regions of the digestive tract of earthworms are colonized by distinct communities of bacteria and fungi that may contribute to the overall microbial community of the compost product [49].

### Dynamics of composting process

We know the thermophilic phase is important to reduce pathogen loads during composting (www.ams.usda.gov/nop). In contrast, the post-thermophilic phase is often ignored. Although recipe was held constant, microbial community composition diversified rapidly through time depending on whether the compost process was managed by windrow, ASP or vermicompost. To our knowledge, this is the first report of temporal dynamics in ASP or vermicompost.

Most of what we know about degradative succession in compost is from windrow studies. Relatively few taxa dominate during the thermophilic phase. For example, we observed that Bacteroidetes dominated at the end of thermophilic phase for windrow, supporting previous work [19]. In contrast, Firmicutes and γ-Proteobacteria were dominant after the thermophilic phase in ASP and vermicompost, respectively. All three of these bacterial phyla contain thermophiles [2]. Furthermore, relative abundance of γ-Proteobacteria, Firmicutes and Actinobactera are reported as indicators of disease suppression [50].

The fungi we observed at the end of thermophilic phase were similar to those reported previously from compost. For example, Dothideomycetes and Eurotiales were both abundant after the thermophilic phase [2], [15]. In contrast to Anastasi et al. [4], we found *Taloromyces* in compost made by all three processes rather than just vermicompost. Other fungi that were relatively abundant after the thermophilic phase have been reported for composts containing ingredients similar to our study. For example, *Cladosporium* has been reported previously on compost based on cattle manure [2]. *Thermomyces* and *Penicillium* have both been isolated from composts containing hardwood bark and manure [2].

Near the end of the composting process, a different and more complex community develops that includes chytrids, protists, Ascomycota, and Stamenopiles [15]. In windrow, Chloroflexi and γ-Proteobacteria decreased in relative abundance during the cooling and curing phases and were surpassed in abundance by Bacteroidetes in finished composts. This supports earlier reports that Bacteroidetes are relatively abundant, and more abundant than γ-Proteobacteria, in finished compost [10], [16]. In contrast, Chloroflexi decreased and abundances of α-Proteobacteria were half those of γ-Proteobacteria in the final product [23]. Actinobacteria become more abundant during the curing phase [51], [52], [53], [54]. Actinobacteria are less likely to be found in vermicompost with the more active microbial communities promoted by earthworms [47].

Numerous mesophilic fungi proliferate during the cooling and curing phases [50]. Many of the fungi we found are known to be widespread saprophytes on soil and dead plant tissue [55]. Similar to Anastasi et al. [4], *Acremonium* and *Cladosporium* occurred in both windrow and vermicompost, and *Scytalidium* was more abundant in windrow than vermicompost. It is no surprise that *Arthrographis* and *Galactomyces* occur in compost given their ability to produce cellulolytic enzymes [56], [57], however, this is the first report for composts made by ASP and vermicompost processes, specifically.

### Conclusion

Microbial communities are abundant and diverse in compost. Communities are organized and influenced by recipe, and post-thermophilic treatment. Composition starts similarly after thermophilic phase and shifts dynamically through time. Economic considerations have driven commercial composters to expedite the composting process. As a result, the focus has been on the effectiveness of the thermophilic phase. The curing phase offers a substrate and climate conducive for microbial recolonization which can be accomplished either by inoculating post-thermophilic compost or preparing a palatable substrate that provides a competitive advantage for colonization by bacteria and fungi that offer biological control, slow-release fertility, and/or promote plant growth. Future research can build on the microbial results presented here to determine which recipe and post-thermophilic phase are best to achieve desired agricultural goals of weed management, disease suppression, and plant growth promotion.

## Supporting Information

Table S1 Median percentage of sequences of the most abundant classified bacteria by compost recipe.
(DOCX)

Table S2 Median percentage of sequences of the most abundant classified bacteria by compost process.
(DOC)
