# Supplementary material for: Changes in Bacterial and Fungal Communities across Compost Recipes, Preparation Methods, and Composting Times
Source: PLoS One. 2013 Nov 21;8(11):e79512. doi: 10.1371/journal.pone.0079512 (PMC3836849; doi:10.1371/journal.pone.0079512)
Supplement: Table S1 — Median percentage of sequences of the most abundant classified bacteria by compost recipe. (DOCX) [file pone.0079512.s001.docx]

Table S1

| Phylum | Class | Order | Family | Manure | Hay | Hardwood | |
| --- | --- | --- | --- | --- | --- | --- | --- |
| Bacteroidetes | Sphingobacteriia | Sphingobacteriales | Flammeovirgaceae | 9.85 | 13.00 | 12.65 | |
| Proteobacteria | Gammaproteobacteria | Other | Other | 3.30 | 2.05 | 1.90 | |
| Bacteroidetes | Sphingobacteriia | Sphingobacteriales | Other | 3.10 | 1.30 | 0.95 | |
| Proteobacteria | Deltaproteobacteria | Myxococcales |  | 2.45 | 2.45 | 2.45 | |
| Bacteroidetes | Other | Other | Other | 2.30 | 2.25 | 1.05 | |
| Proteobacteria | Other | Other | Other | 2.20 | 2.50 | 2.15 | |
| Bacteroidetes | Sphingobacteriia | Sphingobacteriales | Saprospiraceae | 1.90 | 0.80 | 0.15 | |
| Proteobacteria | Alphaproteobacteria | Rhizobiales | Hyphomicrobiaceae | 1.70 | 1.60 | 2.00 | |
| Proteobacteria | Alphaproteobacteria | Rhizobiales | Other | 1.50 | 0.55 | 0.45 | |
| Bacteroidetes | Flavobacteriia | Flavobacteriales | Flavobacteriaceae | 1.40 | 1.75 | 0.15 | |
| Proteobacteria | Betaproteobacteria | Other | Other | 1.35 | 1.30 | 0.70 | |
| Proteobacteria | Alphaproteobacteria | Other | Other | 1.30 | 1.45 | 1.35 | |
| Actinobacteria | Acidimicrobiia | Acidimicrobiales | koll13 | 1.25 | 1.35 | 0.75 | |
| Bacteroidetes | Sphingobacteriia | Sphingobacteriales | Chitinophagaceae | 1.15 | 2.55 | 2.90 | |
| Proteobacteria | Betaproteobacteria | Methylophilales | Other | 1.15 | 0.30 | 0.00 | |
| Proteobacteria | Gammaproteobacteria | Xanthomonadales | Xanthomonadaceae | 1.10 | 1.80 | 0.15 | |
| Proteobacteria | Deltaproteobacteria | Other | Other | 1.00 | 0.85 | 0.55 | |
| Proteobacteria | Gammaproteobacteria | Xanthomonadales | Sinobacteraceae | 1.00 | 4.30 | 3.70 | |
| Planctomycetes | Planctomycetia | Pirellulales | Pirellulaceae | 0.95 | 0.55 | 1.05 | |
| Bacteroidetes | Sphingobacteriia | Sphingobacteriales |  | 0.90 | 1.40 | 0.95 | |
| Chloroflexi | Anaerolineae | SBR1031 | SHA-31 | 0.85 | 0.20 | 0.05 | |
| Planctomycetes | Phycisphaerae | Phycisphaerales |  | 0.85 | 0.45 | 0.25 | |
| Chloroflexi | Anaerolineae | CFB-26 |  | 0.75 | 0.65 | 0.60 | |
| Chloroflexi | Anaerolineae | SBR1031 | A4b | 0.70 | 0.35 | 4.90 | |
| Proteobacteria | Gammaproteobacteria | HTCC2188 | 211ds20 | 0.65 | 0.45 | 0.40 | |
| Gemmatimonadetes | Gemm-5 |  |  | 0.60 | 3.40 | 2.80 | |
| Proteobacteria | Gammaproteobacteria | Alteromonadales | Alteromonadaceae | 0.55 | 1.30 | 0.05 | |
| Verrucomicrobia | Opitutae | Opitutales | Opitutaceae | 0.55 | 0.45 | 0.55 | |
| Firmicutes | Bacilli | Bacillales | Other | 0.50 | 0.70 | 0.30 | |
| Firmicutes | Clostridia | Clostridiales | Clostridiaceae | 0.50 | 0.85 | 0.05 | |
| Spirochaetes | Spirochaetes | Spirochaetales | Spirochaetaceae | 0.50 | 0.45 | 0.00 | |
| Bacteroidetes | Sphingobacteriia | Sphingobacteriales | Sphingobacteriaceae | 0.45 | 1.50 | 0.15 | |
| Chloroflexi | Anaerolineae | GCA004 |  | 0.40 | 0.05 | 1.20 |  |
| Proteobacteria | Betaproteobacteria | Burkholderiales | Comamonadaceae | 0.40 | 0.35 | 0.20 |  |
| Acidobacteria | Acidobacteria-6 | iii1-15 |  | 0.35 | 0.10 | 1.30 |  |
| Bacteroidetes | Sphingobacteriia | Sphingobacteriales | Flexibacteraceae | 0.35 | 0.40 | 0.05 |  |
| Actinobacteria | Actinobacteria | Actinomycetales | Other | 0.30 | 1.40 | 1.45 |  |
| Verrucomicrobia | [Spartobacteria] | [Chthoniobacterales] | [Chthoniobacteraceae] | 0.15 | 0.35 | 1.40 |  |
| Actinobacteria | Actinobacteria | Actinomycetales | Streptosporangiaceae | 0.05 | 0.80 | 1.50 |  |

S2. Median percentage of sequences of the most abundant classified bacteria by compost process. Sorted by windrow.

| Phylum | Class | Order | Family | windrow | ASP | vermi |
| --- | --- | --- | --- | --- | --- | --- |
| Bacteroidetes | Sphingobacteriia | Sphingobacteriales | Flammeovirgaceae | 13.23 | 15.78 | 13.93 |
| Chloroflexi | Anaerolineae | SBR1031 | A4b | 6.45 | 2.85 | 0.48 |
| Chloroflexi | Anaerolineae | GCA004 |  | 5.13 | 6.15 | 0.18 |
| Proteobacteria | Gammaproteobacteria | Xanthomonadales | Sinobacteraceae | 3.63 | 6.28 | 1.53 |
| Chlorobi | Ignavibacteria | Ignavibacteriales | Ignavibacteriaceae | 3.30 | 1.45 | 0.03 |
| Chloroflexi | Anaerolineae | Other | Other | 2.58 | 0.38 | 0.23 |
| Proteobacteria | Deltaproteobacteria | Myxococcales |  | 2.53 | 2.53 | 2.53 |
| Bacteroidetes | Other | Other | Other | 2.15 | 1.50 | 2.40 |
| Proteobacteria | Betaproteobacteria | MND1 |  | 1.98 | 1.18 | 0.20 |
| Proteobacteria | Gammaproteobacteria | Other | Other | 1.75 | 2.63 | 3.38 |
| Proteobacteria | Other | Other | Other | 1.48 | 2.00 | 2.15 |
| Bacteroidetes | Sphingobacteriia | Sphingobacteriales | Rhodothermaceae | 1.40 | 1.53 | 0.10 |
| Proteobacteria | Deltaproteobacteria | Syntrophobacterales | Syntrophobacteraceae | 1.33 | 0.65 | 0.10 |
| Acidobacteria | Acidobacteria-6 | iii1-15 |  | 1.20 | 0.95 | 0.65 |
| Proteobacteria | Alphaproteobacteria | Rhizobiales | Hyphomicrobiaceae | 1.15 | 1.45 | 1.53 |
| Bacteroidetes | Sphingobacteriia | Sphingobacteriales | Other | 1.10 | 1.55 | 1.45 |
| Acidobacteria | Solibacteres | Solibacterales | Solibacteraceae | 1.03 | 0.40 | 0.93 |
| Proteobacteria | Alphaproteobacteria | Other | Other | 1.00 | 0.75 | 1.78 |
| Proteobacteria | Gammaproteobacteria | Xanthomonadales |  | 0.98 | 1.40 | 0.48 |
| Bacteroidetes | Sphingobacteriia | Sphingobacteriales | Chitinophagaceae | 0.93 | 2.35 | 2.98 |
| Planctomycetes | Planctomycetia | Pirellulales | Pirellulaceae | 0.90 | 0.78 | 1.18 |
| Proteobacteria | Betaproteobacteria | Other | Other | 0.78 | 0.55 | 1.35 |
| Gemmatimonadetes | Gemm-5 |  |  | 0.73 | 1.45 | 0.15 |
| Proteobacteria | Betaproteobacteria | IS-44 |  | 0.68 | 0.75 | 0.65 |
| Proteobacteria | Deltaproteobacteria | Other | Other | 0.53 | 1.43 | 3.48 |
| Proteobacteria | Gammaproteobacteria | Pseudomonadales | Pseudomonadaceae | 0.48 | 0.28 | 1.38 |
| Bacteroidetes | Flavobacteriia | Flavobacteriales | Flavobacteriaceae | 0.20 | 0.88 | 2.13 |
| Proteobacteria | Gammaproteobacteria | HTCC2188 | 211ds20 | 0.20 | 0.50 | 2.03 |
